# Development of a MRI-Based Radiomics Nomogram for Prediction of Response of Patients With Muscle-Invasive Bladder Cancer to Neoadjuvant Chemotherapy

**DOI:** 10.3389/fonc.2022.878499

**Published:** 2022-05-11

**Authors:** Xinxin Zhang, Yichen Wang, Jin Zhang, Lianyu Zhang, Sicong Wang, Yan Chen

**Affiliations:** ^1^ Department of Diagnostic Radiology, National Cancer Center/National Clinical Research Center for Cancer/Cancer Hospital, Chinese Academy of Medical Sciences and Peking Union Medical College, Beijing, China; ^2^ Magnetic Resonance Imaging Research, General Electric Healthcare, Beijing, China

**Keywords:** muscle-invasive bladder cancer, neoadjuvant chemotherapy, MRI, radiomics, nomogram

## Abstract

**Objective:**

To develop and evaluate the performance of a magnetic resonance imaging (MRI)-based radiomics nomogram for prediction of response of patients with muscle-invasive bladder cancer (MIBC) to neoadjuvant chemotherapy (NAC).

**Methods:**

A total of 70 patients with clinical T2-4aN0M0 MIBC were enrolled in this retrospective study. For each patient, 1316 radiomics features were extracted from T2-weighted images (T2WI), diffusion-weighted images (DWI), and apparent diffusion coefficient (ADC) maps. The variance threshold algorithm and the Student’s t-test or the Mann–Whitney U test were applied to select optimal features. Multivariate logistic regression analysis was used to eliminate irrelevant features, and the retained features were incorporated into the final single-modality radiomics model. Combined radiomic models were generated by combining single-modality radiomics models. A radiomics nomogram, incorporating radiomics signatures and independent clinical risk factors, was developed to determine whether the performance of the model in predicting tumor response to NAC could be further improved.

**Results:**

Based on pathological T stage post-surgery, 36 (51%) patients were classified as good responders (GR) and 34 (49%) patients as non-good responders (non-GR). In addition, 3 single-modality radiomics models and 4 combined radiomics models were established. Among all radiomics models, the combined radiomics model based on T2WI_Score, DWI_Score, and ADC_Score yielded the highest area under the receiver operating characteristics curve (AUC) (0.967, 95% confidence interval (CI): 0.930–0.995). A radiomics nomogram, integrating the clinical T stage and 3 single-modality radiomics models, yielded a higher AUC (0.973, 95%CI: 0.934–0.998) than other combined radiomics models.

**Conclusion:**

The proposed MRI-based radiomics nomogram has the potential to be used as a non-invasive tool for the quantitatively prediction of tumor response to NAC in patients with MIBC.

## Introduction

Neoadjuvant chemotherapy (NAC), as the standard treatment for muscle-invasive bladder cancer (MIBC), can significantly improve overall survival (OS) (1, 2). However, only 29–55% of patients with MIBC have shown a favorable response to NAC (3, 4). Non-responders are unnecessarily exposed to treatment-related adverse effects, and delay in definitive surgical treatment may have profound effects on OS (5). Therefore, accurate identification of tumor response to NAC is critical to develop more efficacious therapeutic strategies.

Some studies have shown that the mutations in single or multiple genes and distinct molecular subtypes of bladder cancer were associated with the efficacy of NAC for bladder cancer (6–9). However, genetic testing and genomic clustering analysis are invasive and expensive. Moreover, it is difficult to evaluate the potential benefits of integrating these biomarkers into decision-making on the use of NAC. While in the radiology, magnetic resonance imaging (MRI), as a reproducible and non-invasive examination method, possesses extensive clinical applications and unique advantages in predicting the efficacy of treatment (10–13). Previously, diffusion-weighted MRI (DW-MRI) has been used for the prediction of therapeutic responses of patients with MIBC noninvasively (14, 15). However, the above-mentioned studies have only concentrated on a single-modality with limited imaging data, therefore, multi-parameter medical imaging studies need to be conducted.

To date, with advances in high-throughput post-processing techniques, radiomics, combining several quantitative imaging features, has exhibited significant advantages in providing more valuable information about the tissue characteristics compared with visual inspection (16, 17). Radiomics, a noninvasive and reliable method, has been extensively used in oncology to improve disease diagnosis and to predict tumor response to treatment in rectal cancer, breast cancer, and cervical cancer (18–21). Nonetheless, the clinical value of radiomics in MIBC patients has still remained elusive. Therefore, the present study aimed to investigate the capability of MRI-based radiomics models to predict response of patients with MIBC to NAC.

## Materials and Methods

### Patients

**Figure 1:**
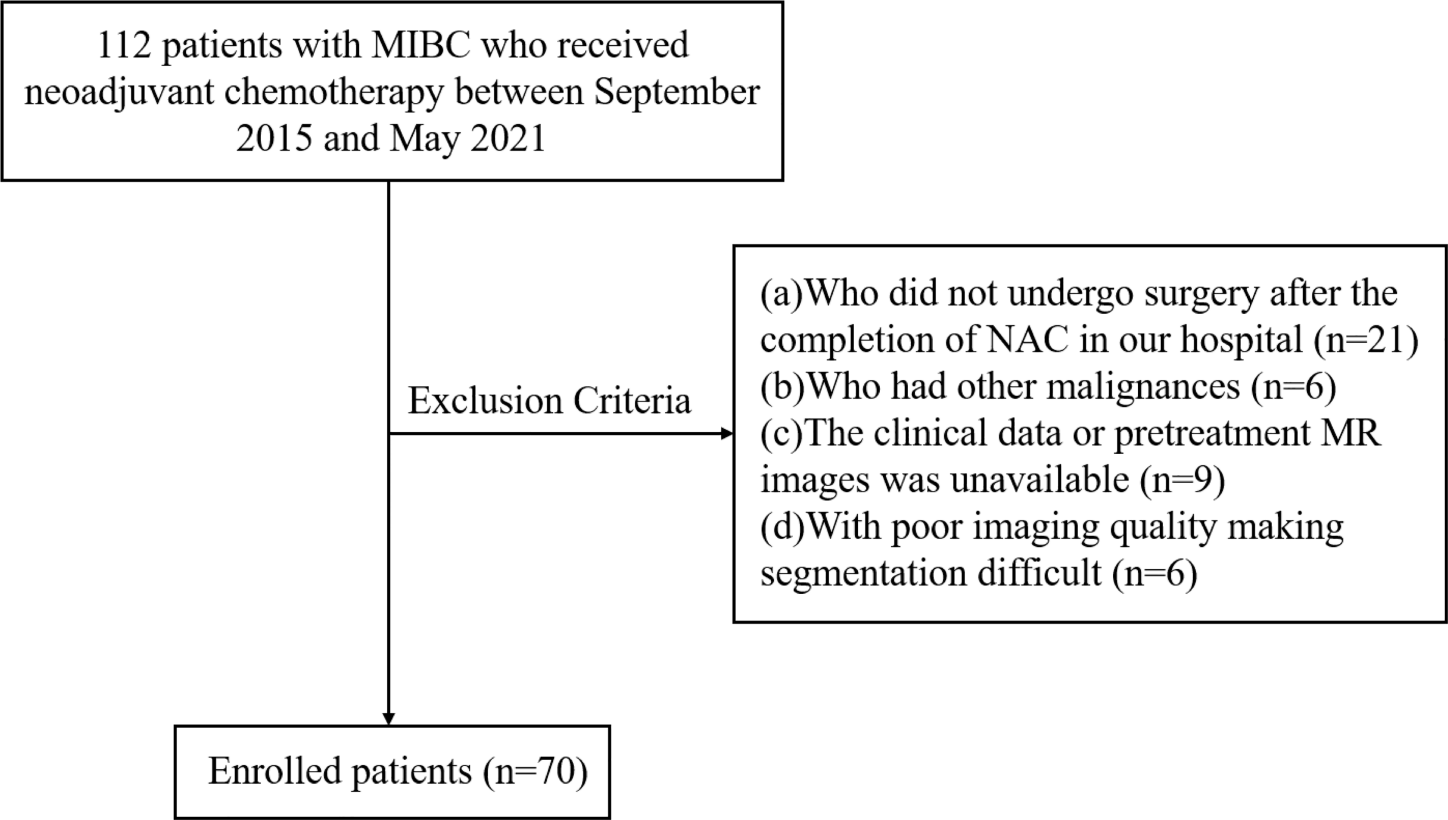
Flowchart of study design. MIBC, muscle-invasive bladder cancer.

We enrolled 112 consecutive patients with MIBC who were treated with NAC in our center between September 2015 and May 2021. The study flowchart is shown in **Figure 1**. The inclusion criteria were as follows: (a) urothelial carcinoma confirmed by biopsy, (b) clinical stage T2-4aN0M0, (c) patients who underwent NAC followed by surgery, (d) patients who received MRI of bladder before NAC and biopsy. The exclusion criteria were as follows: (a) patients who did not undergo surgery after the completion of NAC in our hospital (n=21), (b) patients who had other malignancies (n=6), (c) unavailability of clinical data or pretreatment MR images (n=9), (d) poor imaging quality making difficulties in segmentation (n=6). Finally, a total of 70 patients were included in the present study. Baseline clinical data, including age, gender, pretreatment histologic grade, number of lesions, clinical T stage (cT), pathological T stage (pT), and treatment were derived from medical records.

### Treatment Protocol and Therapeutic Effects

All patients received 2-4 cycles of gemcitabine/cisplatin-based NAC, which was consisted of 1,000 mg/m^2^ gemcitabine on days 1 and 8 and 75 mg/m^2^ cisplatin on day 2 *via* intravenous infusion every 21 days. After NAC, surgery was performed according to the restaging of the remaining tumor, which was evaluated by MRI of the bladder. Patients with non-MIBC (≤ cT1) received transurethral resection of bladder tumor (TURBT) and concurrent chemoradiotherapy. Patients with MIBC (≥ cT2) underwent partial or radical cystectomy with pelvic lymphadenectomy.

The evaluation of tumor response to NAC was carried out according to the pT stage after surgery. Patients with non-MIBC (≤ pT1) were categorized as good responders (GR), while those with MIBC (≥ pT2) were categorized as non-good responders (non-GR).

### MRI

All patients underwent MRI with eight-channel phased array body coil through a 3.0-T scanner (GE Discovery 750; GE Healthcare, Milwaukee, WI, USA). T2-weighted images (T2WI), DW images (DWI), and apparent diffusion coefficient (ADC) maps derived from DWI were obtained. Further imaging data are presented in **Table 1**.

**Table 1:** MRI sequence parameters.

Parameters	Axial T2WI	Sagittal T2WI	DWI
Repetition time (ms)	5043	6240	2288
Echo time (ms)	102	102	58.4
No. of echo trains per section	21	21	1
Matrix size	320×256	320×256	128×160
Field of view (cm×cm)	20×20	22×22	38×38
Slice thickness (mm)	3	3	5
Interslice gap (mm)	0.3	0.3	0.3
Number of excitations	2	1	4
Acquisition time (sec)	146	182	32
b-value (sec/mm^2^)			0, 1000

### Tumor Segmentation and Feature Extraction

Image segmentation was performed by two radiologists independently (reader 1 and reader 2 with 3 and 23 years of experience in interpreting genitourinary MR images, respectively). These two radiologists were blinded to patients’ clinical data, while they had access to all MR images to verify the lesion boundaries and exclude areas of necrosis or vessels. For patients with multiple lesions, the one with the highest clinical T stage or the largest tumor with the equal T stage was selected for analysis. Regions of interest (ROIs) were manually delineated slice-by-slice through the whole tumor on T2WI and DWI (b value of 1000 s/mm^2^) sequences, respectively, using ITK-SNAP (www.itksnap.org). Contours of ROIs delineated on DWI were saved and imported into the corresponding ADC maps.

Radiomics features were extracted automatically using the Artificial Intelligence Kit software (ver. 3.3.0; A.K., GE Healthcare) based on the open-source Pyradiomics python package. A total of 1316 radiomics features were extracted from T2WI, DWI, and ADC maps of each patient, which included 18 first-order histogram features, 14 shape-based features, 24 gray-level co-occurrence matrix (GLCM) features, 16 gray-level size zone matrix (GLSZM) features, 16 gray-level run length matrix (GLRLM) features, 14 gray-level dependence matrix (GLDM) features, 744 wavelet features, 5 neighboring gray-tone difference matrix (NGTDM) features, 186 Laplacian of Gaussian (LoG_sigma=2.0/3.0_) features, and 279 local binary pattern features.

The interclass correlation coefficients (ICCs) were calculated to investigate the consistency of features derived from ROIs drawn by two radiologists. Those stable features with ICCs ≥ 0.75 were applied for the subsequent feature selection process. Before the feature selection, data preprocessing and feature normalization were performed. When the data exceeded the range of the mean value and standard deviation, the outliers were replaced by the median of the specific variance vector.

### Selection of Radiomics Features and Construction of Radiomics Models

The feature selection was performed within each modality of the T2WI, DWI, and ADC maps. Firstly, the variance threshold algorithm (variance threshold selected at 1.0, so that features with variance ≥ 1.0 were selected) was applied for dimensionality reduction. Secondly, we used the Student’s t-test or the Mann–Whitney U test to eliminate irrelevant features, and features with P <0.05 were selected. Finally, the multivariate logistic regression analysis was used to select the most significant features. The radiomics scores (Radscore), which included the T2WI_Score, DWI_Score, and ADC_Score, were calculated *via* the linear combination of the selected features weighted by their respective coefficients from each patient.

The combined radiomics models were established by combining single-modality radiomics models with all possible combinations using multivariable logistic regression. The performance of each radiomics model in predicting tumor response to NAC was evaluated by the receiver operating characteristic (ROC) curve analysis. The DeLong test was used to compare differences among radiomics models.

### Nomogram Construction

The differences in clinical factors between GR and non-GR groups were compared using univariate analysis. The radiomics nomogram was constructed by integrating the independent clinical risk factors and Radscore of the single-modality radiomics model. The predictive performance of the nomogram was estimated based on the area under the receiver operating characteristics curve (AUC), accuracy, specificity, and sensitivity. Afterwards, the calibration curve was used to depict the performance-associated characteristics of the radiomics models graphically. Finally, decision curve analysis was performed to evaluate the clinical applicability of multimodal radiomics by quantifying the net benefit at different threshold probabilities. The DeLong test was used to compare differences among the radiomics models and the nomogram.

### Statistical Analysis

The statistical analysis was performed using R 3.4.3 software (R Core Team, Vienna, Austria). The independent t-test or the Mann-Whitney U test was used for the analysis of continuous variables. Categorical variables were assessed by the Chi-square test or the Fisher’s exact test, followed by Bonferroni correction. A two-tailed P < 0.05 was considered statistically significant.

## Results

### Patients’ Characteristics

The relevant clinical characteristics of patients are summarized in **Table 2**. In our study, there were 36 (51%) and 34 (49%) patients in the GR and non-GR groups, respectively. Only cT stage was significantly different between the GR and non-GR groups (P-adjusted < 0.0167). Other clinical characteristics were not statistically different between the GR and non-GR groups (P = 0.28–0.99).

**Table 2:** Characteristics of patients with MIBC.

Characteristics	No. of patients	Good Responder (n = 36)	Non-Good Responder (n = 34)	P value
Age* (years old, mean ± SD)	61.9 ± 8.3	61.1 ± 9.1	62.8 ± 7.4	0.39
Gender				
Male	62	30 (83%)	32 (94%)	0.30
Female	8	6 (17%)	2 (6%)	
Number of lesions				0.99
Solitary	48	24 (64%)	24 (60%)	
Multiple	22	12 (36%)	10 (40%)	
Clinical T stage (cT)				0.004
cT2	21	17 (55%)	4 (28%)	
cT3	43	17(39%)	26 (64%)	
cT4a	6	2 (6%)	4 (8%)	
Histological grade				0.28
Low grade	14	9 (36%)	5 (36%)	
High grade	56	27 (64%)	29 (64%)	
NAC courses				0.38
2	18	7 (19%)	11 (32%)	
3	32	19 (53%)	13 (38%)	
4	20	10 (28%)	10 (30%)	
Surgery				N/A
Radical cystectomy + PLND	23	1 (12%)	22 (60%)	
Partial cystectomy + PLND	9	3 (21%)	6 (12%)	
TURBT+CRT	38	32 (67%)	6 (28%)	
Pathological Stages (pT)				N/A
pT0	2	2 (5%)		
pTa	1	1 (3%)		
pTis	1	1 (3%)		
pT1	32	32 (89%)		
pT2	13		13 (38%)	
pT3	15		15 (44%)	
pT4	6		6 (18%)	

Except where indicated, data include number of participants, with percentages in parentheses. SD, standard deviation, MIBC,muscle-invasive bladder cancer; TURBT,transurethral resection of bladder tumor; NAC,neoadjuvant chemotherapy; CRT, chemoradiotherapy; PLND,pelvic lymphadenectomy; N/A,not applicable; RC, radical cystectomy.

*Data are presented as mean ± standard deviation.

### Selection of Radiomics Features and Construction of Radiomics Models

After feature selection, three optimal radiomics features were retained from the T2WI, DWI, ADC maps, respectively. The selected features for each single-modality radiomics signature with non-zero coefficients are presented in the Radscore calculation formula. The single-modality radiomics signature was established with a Radscore calculated as follows:


$$\begin{array}{l} \text{T}2\text{WI}\_\text{score}=\;0.13\;+\;(1.40\\ \;\times \text{ log}\_\text{sigma}\_3\_0\_\text{mm}\_3\text{D}\_\text{NGTDM}\_\text{Contrast}+\;1.17\\ \;\times \text{wavelet}\_\text{LowHighHigh}\_\text{GLDM}\_\\ \text{DependenceVariance }–\;1.00\;\times \text{ wavelet}\_\text{HHH}\_\text{NGTDM})\\ \text{DWI}\_\text{score }=\;0.12+(-0.56\\ \;\times \text{wavelet}\_\text{ LowHighHigh }\_\text{GLCM}\_\text{Correlation }+\;0.76\\ \;\times \text{ wavelet}\_\text{LowHighHigh }\_\text{firstorder}\_\text{Entropy }–\;0.66\\ \;\times \text{ wavelet}\_\text{LowLowLow}\_\text{GLCM}\_\text{Correlation})\\ \text{ ADC}\_\text{score }=\;0.18\;+\;(0.94\\ \;\times \text{ wavelet}\_\text{HighLowLow}\_\text{GLRLM}\_\text{RunEntropy }+\;0.82\\ \;\times \text{wavelet}\_\text{LowLowLow}\_\text{firstorder}\_10\text{Percentile }+\;0.70\\ \;\times \text{wavelet}\_\text{LowLowHigh}\_\text{GLCM}\_\text{DifferenceAverage}) \end{array}$$

The combined radiomics models were constructed by integrating different single-modality radiomics models derived from each modality (T2WI, DWI, and ADC maps) using the following formulas:


$$\begin{array}{l} {\text{Model}}_{\text{T}2\text{WI}+\text{DWI}}=–0.12+\left(0.94\times \text{ Radscore}\_\text{T}2\text{WI }+0.78\;\times \text{Radscore}\_\text{DWI}\right)\\ {\text{Model}}_{\text{T}2\text{WI}+\text{ADC}}=0.05\;+\;\left(1.16\;\times \text{ Radscore}\_\text{T}2\text{WI }+\;1.49\;\times \text{Radscore}\_\text{ADC}\right)\\ {\text{Model}}_{\text{DWI}+\text{ADC}}=0.01+\left(0.95\;\times \text{ Radscore}\_\text{DWI }+\;1.00\;\times \text{Radscore}\_\text{ADC}\right)\\ {\text{Model}}_{\text{T}2\text{WI}+\text{DWI}+\text{ADC}}=\;0.02\;+\;(1.19\;\times \text{ Radscore}\_\\ \text{T}2\text{WI }+\;1.01\;\times \text{ Radscore}\_\text{DWI}+\;1.69\;\times \text{ Radscore}\_\text{ADC}) \end{array}$$

### Diagnostic Performance of the Radiomics Models

**Figure 2:**
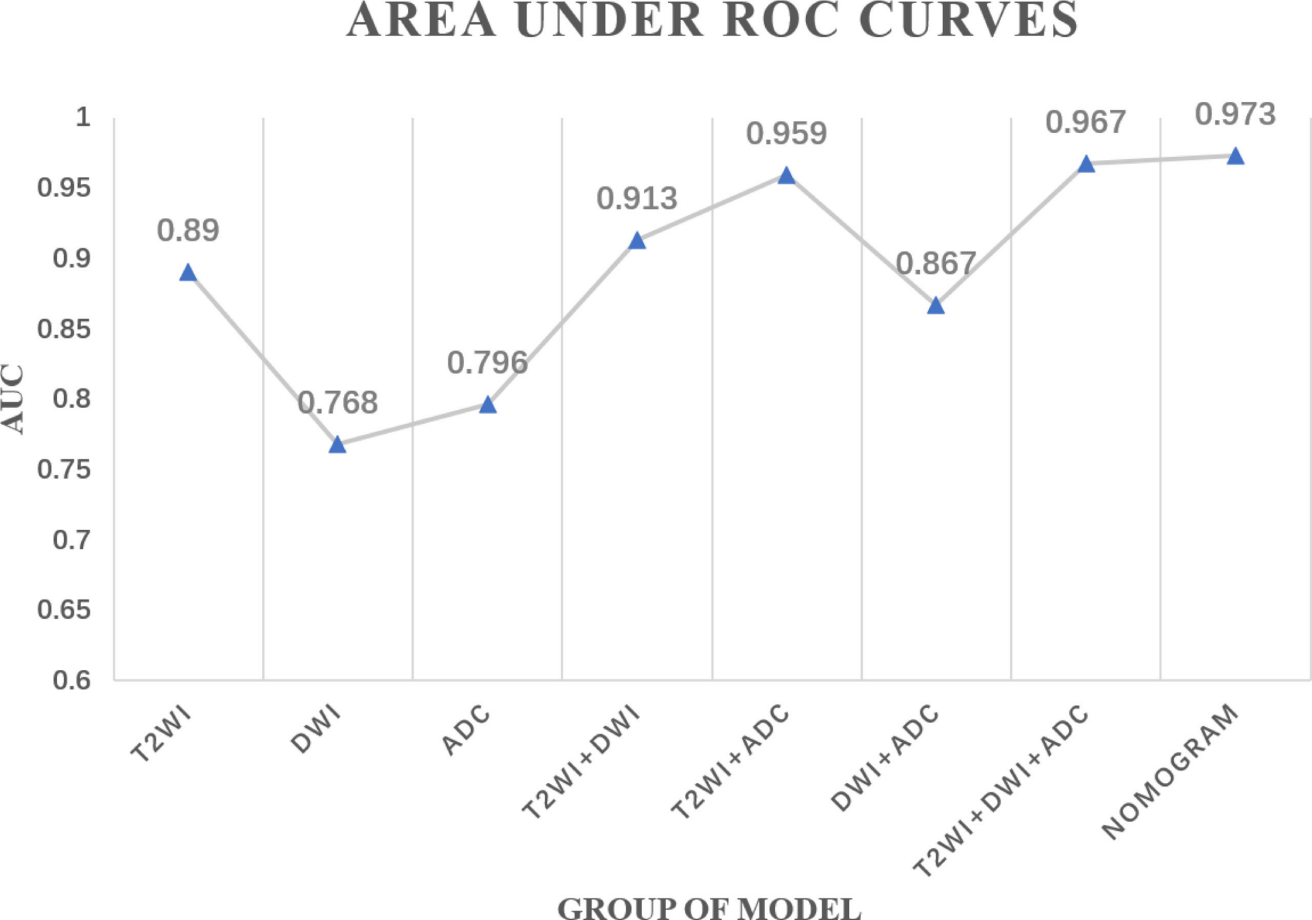
Area under the receiver operating characteristics curves of each radiomics model.

In total, three single-modality radiomics models and four combined radiomics models were established. The performances of all the radiomics models in predicting the tumor response to NAC are shown in **Table 3** and **Figure 2**. Among single-modality radiomics models, T2WI yielded a higher AUC of 0.890 [95% confidence interval (CI), 0.639–0.831] than others. The combined radiomics model of Radscore_T2WI, Radscore_DWI, and Radscore_ADC (Model_T2WI+DWI+ADC_) yielded the highest AUC, sensitivity, and specificity of 0.967 (95% CI, 0.930–0.995), 0.889, and 0.941, respectively, for predicting the tumor response to NAC.

**Table 3:** Diagnostic performance of all radiomics models and the nomogram.

	AUC (95%CI)	Sensitivity	Specificity	PPV	NPV	Accuracy
Model_T2WI_	0.890 (0.639,0.831)	0.639	0.971	0.958	0717	0.800
Model_DWI_	0.768 (0.619,0.803)	0.861	0.618	0.705	0.808	0.743
Model_ADC_	0.796 (0.694,0.867)	0.806	0.676	0.906	0.667	0.743
Model_T2WI+DWI_	0.913 (0.648,0.832)	0.694	0.971	0.962	0.750	0.829
Model_T2WI+ADC_	0.959 (0.684,0.866)	0.861	0.941	0.939	0.865	0.900
Model_DWI+ADC_	0.867 (0.712,0.871)	0.861	0.765	0.795	0.839	0.814
Model_T2WI+DWI+ADC_	0.967 (0.930,0.995)	0.889	0.941	0.941	0.889	0.914
Model_nomogram_	0.973 (0.934,0.998)	0.944	0.941	0.944	0.941	0.943

AUC, area under the receiver operating characteristic curve; CI, confidence interval; PPV, positive-predictive value; NPV, negative-predictive value; cT, clinical T stage.

### Development and Performance of the Radiomics Nomogram

**Figure 3:**
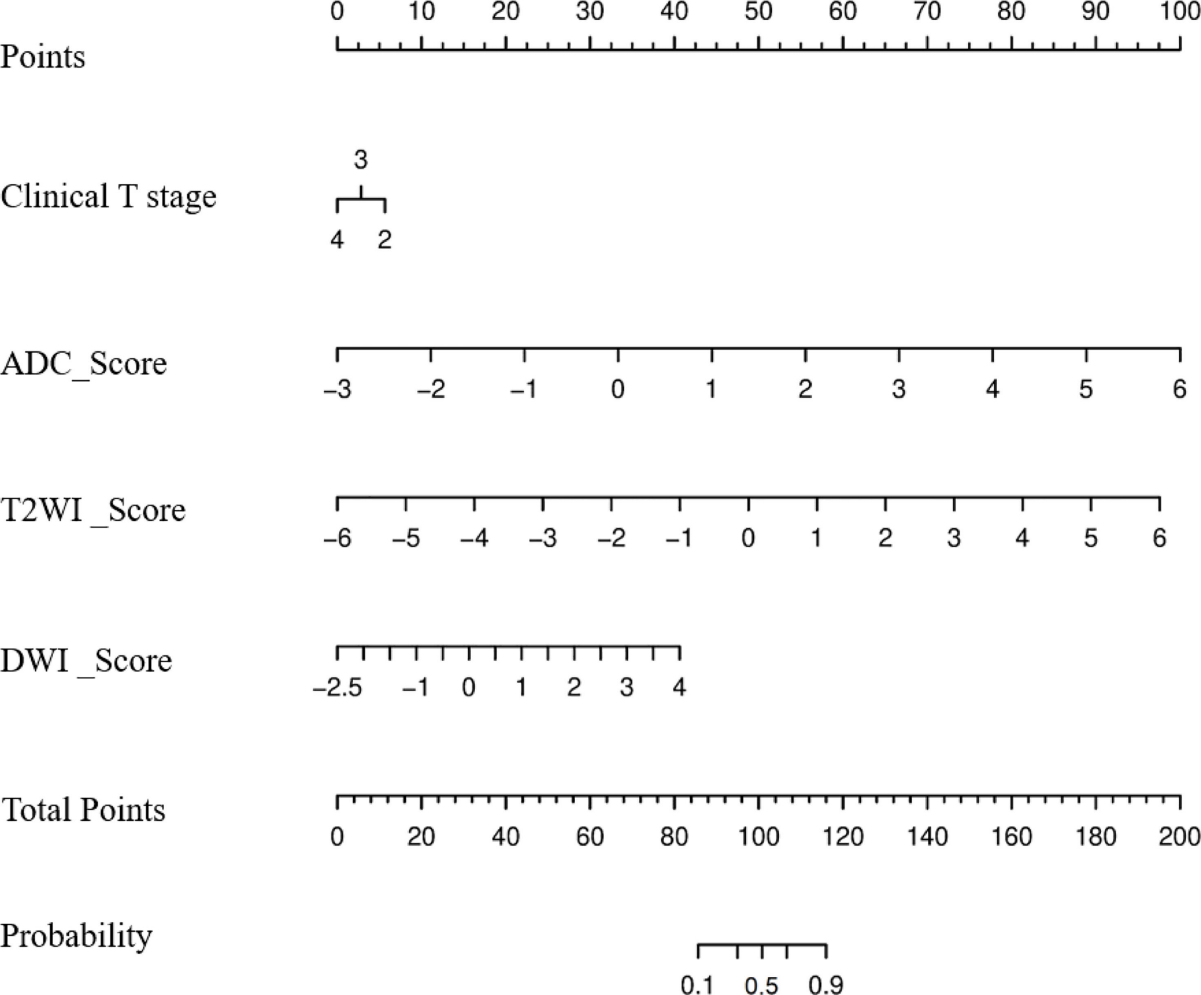
Nomogram to predict the probability of becoming a good responder after neoadjuvant chemotherapy.

A radiomics nomogram was constructed by integrating the clinical T stage and Radscore_T2WI, Radscore_DWI, and Radscore_ADC using the following formula (**Figure 3**):


$${\text{Model}}_{\text{nomogram}}=–1.92\;+\;(1.60\;\times \text{clinical}\_\text{T}\_\text{stage }+\;1.33\times \text{ Radscore}\_\text{T}2\text{WI}+\;0.69\;\times \text{ Radscore}\_\text{DWI }+\;1.53\;\times \text{ Radscore}\_\text{ADC})$$

**Figure 4:**
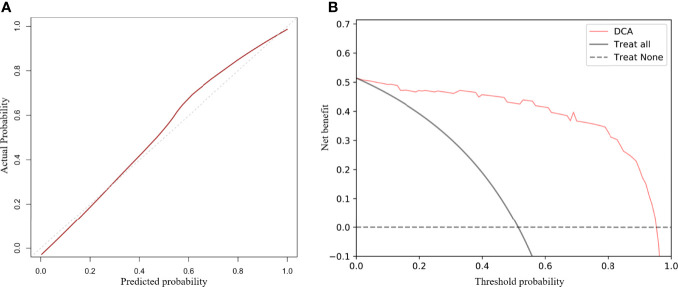
Calibration curves and decision curve analysis (DCA) of the nomogram. Calibration curves for the radiomics nomogram **(A)**. The Y-axis represents actual outcome of response to neoadjuvant chemotherapy, and the X-axis represents the predicted probability. The closer the fit of the diagonal red line to the ideal dotted line indicates the predictive accuracy of the nomogram. DCA for the radiomics nomogram **(B)**. The Y-axis represents the net benefit. The X-axis represents the threshold probability. The net benefit of the nomogram is greater than that of the all or none scheme at a wide range of threshold probabilities.

The calibration curve of the radiologic nomogram showed a good agreement between the probability of tumor response to NAC assessed by the nomograms and the actual probability (**Figure 4**). The Hosmer–Lemeshow test yielded a non-significant result (P = 0.785), which indicated no departure from the goodness of fit. The nomogram yielded AUC, sensitivity, and specificity of 0.973 (95% CI: 0.934–0.998), 0.944, and 0.941, respectively, for predicting the tumor response to NAC (**Table 2**). The results of Decision curve analysis indicated that when the threshold probability was between 0 and 0.96, the net benefit of using the radiomics model to predict the tumor response to NAC was greater than that of the all or none scheme (**Figure 4**).

## Discussion

Noninvasively predicting tumor response to NAC in patients with MIBC is of great significance. In previous studies, MRI had achieved good performances in predicting tumor response to treatment (10, 14). Yoshida et al. reported that mean ADC value (before treatment) was a potential biomarker for predicting pathological complete response (pCR) of MIBC patients to chemoradiotherapy (10). Nguyen et al. showed that histogram analysis of ADC value may provide valuable information to predict the response to NAC prior to the treatment (14). Although these results indicated that ADC value had a great potential in predicting treatment response, their limitations lay in the small sample size and limited imaging information. Therefore, further research and exploration are needed.

As a new non-invasive technique, radiomics can provide more information by extracting high-throughput features from medical images. However, few previous studies have shown that radiomics features considerably depend on various MR acquisition/reconstruction scenarios and pre-processing steps (i.e., normalization and quantization) (22, 23). To avoid the influences of these factors, we chose MR images from the same MR scanner with the same scanning parameters. In the present study, the pretreatment radiomics nomogram, incorporating clinical T stage with the single-modality radiomics model, had a higher predictive capability than the radiomic models alone for prediction of response of patients with MIBC to NAC.

We used the radiomics approach to extract a large number of quantitative features from T2WI, DWI, and ADC maps. Of the optimal features selected, wavelet-transformed features accounted for the majority, which was consistent with previous studies (24, 25). Zhao et al. demonstrated that most features selected in their radiomics nomogram were wavelet-transformed features (24). Another study showed that wavelet-transformed textures improved the performance of the model in predicting response of patients with breast cancer to NAC (25). These studies revealed that wavelet-transformed features may be the key component in radiomic models.

In our study, a significant difference was found in cT stage between GR and non-GR groups. Lesions with cT2 stage accounted for a larger proportion than lesions with cT3 stage in the GR group, while a larger proportion of lesions with cT3 stage was found in the non-GR group. This indicated that tumors with a lower cT stage were more likely to have a good response to NAC.

We investigated differences in the performance of combined radiomics models and single-modality radiomics models. The Model_T2WI_ achieved the best predictive performance with the highest AUC among the single-modality radiomics models. This revealed that the T2WI sequence might have a preferable effect on predicting tumor response to NAC, which was similar to the findings of previous studies (26, 27). Among combined radiomics models, Model_T2WI+DWI+ADC_ based on anatomical and functional imaging exhibited a better predictive performance than single-modality radiomics models (28). The nomogram, which combined the clinical T stage with Radscores, preserved the significant features of each model and yielded the highest AUC in predicting tumor response to NAC. The calibration curves further highlighted the accuracy of the prediction performance. The DCA clearly showed that the nomogram may provide a greater net benefit than the “treat all” or “treat none” strategies.

In the era of precision medicine, the accuracy of prediction can only be improved *via* comprehensively analyzing all useful information. Previous studies have shown that molecular subtype, mRNA expression analysis, and gene mutations have the potential to predict pCR of MIBC patients to NAC (8, 9, 29). However, the performance of biomarkers in predicting the efficacy of NAC is not promising. Moreover, the use of biomarkers to predict the efficacy of NAC for MIBC patients has still some limitations. Genetic testing and genomic clustering analysis are invasive, expensive, and subjected to limitations of tumor biopsy samples. To date, no biomarkers have been incorporated into routine clinical practice. Importantly, this study is the first attempt to explore the nomogram incorporating high-throughput MRI-based radiomics features with clinical T stage for individualized prediction of response of MIBC patients to NAC. The nomogram achieved a favorable performance in quantitatively predicting tumor response to NAC. The nomogram may assist clinicians to make clinical decisions and develop further effective treatments. Based on this nomogram, for those patients who might not have a good response to NAC, radical cystectomy with lymph node dissection would be preferred (30).

## Limitations

There are several limitations in the present study. Firstly, due to the small sample size, no validation of the model using a separate cohort was performed, which might cause overestimation of the predictive performance of the radiomics nomogram. Secondly, all the MR images were acquired from a single institution with the same scanner. Thirdly, for cases with multiple tumors, we only analyzed the lesion with the highest clinical T stage or the largest one with the same T stage, which might lead to selection bias. Hence, further multicenter study with a larger sample size is required, and it is essential to indicate whether MRI-based radiomics can predict the OS of patients with MIBC undergoing NAC.

## Conclusions

The proposed MRI-based radiomics nomogram, integrating radiomics features with MRI-determined cT stage, has the potential to accurately predict tumor response to NAC and it may enable clinicians to optimize clinical decision-making in patients with MIBC.

## Data Availability Statement

The original contributions presented in the study are included in the article/supplementary material. Further inquiries can be directed to the corresponding author.

## Ethics Statement

The studies involving human participants were reviewed and approved by Institutional Review Board of the Cancer Hospital, Chinese Academy of Medical Sciences. Written informed consent for participation was not required for this study in accordance with the national legislation and the institutional requirements.

## Author Contributions

Among the authors in the list, XZ has done a lot of work in research design, data collection, paper writing modification, and paper finalization. YW and JZ not only participated in the data collection and writing of the paper. LZ made great efforts in the design and drafting of this paper. SW made some contributions to the data analysis and writing of the article. YC has done a lot of work in research design, data analysis, and paper finalization. All authors contributed to the article and approved the submitted version.

## Funding

This work was supported by the Beijing Council of Science and Technology [Grant Number Z181100001718089]. The funding source is not involved in study design in the collection, analysis, and interpretation of data, in the writing of the report, and in the decision to submit the article for publication.

## Conflict of Interest

SW is an employee of GE Healthcare.

The remaining authors declare that the research was conducted in the absence of any commercial or financial relationships that could be construed as a potential conflict of interest.

## Publisher’s Note

All claims expressed in this article are solely those of the authors and do not necessarily represent those of their affiliated organizations, or those of the publisher, the editors and the reviewers. Any product that may be evaluated in this article, or claim that may be made by its manufacturer, is not guaranteed or endorsed by the publisher.

## Abbreviations

ADC, apparent diffusion coefficient; AUC, area under the curve; CI, confidence interval; DWI, diffusion-weighted imaging; cT, clinical T stage; GLCM, gray-level co-occurrence matrix features; GLDM, gray-level dependence matrix features; GLRLM, gray-level run length matrix features; GR, good responder; MIBC, muscle-invasive bladder cancer; NAC, neoadjuvant chemotherapy; non-GR, non-good responder; NGTDM, neighboring gray-tone difference matrix features; pCR, pathological complete response; ROI, region of interest; TURBT, transurethral resection of bladder tumor; T2WI, T2-weighted images; pT, pathological T stage.
